# Circadian clock function does not require the histone methyltransferase MLL3

**DOI:** 10.1096/fj.202200368R

**Published:** 2022-06-15

**Authors:** Matthew Baxter, Toryn Poolman, Peter Cunningham, Louise Hunter, Maria Voronkov, Gareth B. Kitchen, Laurence Goosey, Nicola Begley, Danielle Kay, Abby Hespe, Robert Maidstone, Andrew S. I. Loudon, David W. Ray

**Affiliations:** ^1^ NIHR Oxford Biomedical Research Centre John Radcliffe Hospital Oxford UK; ^2^ Oxford Centre for Diabetes, Endocrinology and Metabolism University of Oxford Oxford UK; ^3^ Centre for Biological Timing Faculty of Biology, Medicine and Health University of Manchester Manchester UK

**Keywords:** circadian, clock, epigenetics, histone, inflammation, KMT2C, methyltransferase, MLL3

## Abstract

The circadian clock controls the physiological function of tissues through the regulation of thousands of genes in a cell‐type‐specific manner. The core cellular circadian clock is a transcription–translation negative feedback loop, which can recruit epigenetic regulators to facilitate temporal control of gene expression. Histone methyltransferase, mixed lineage leukemia gene 3 (MLL3) was reported to be required for the maintenance of circadian oscillations in cultured cells. Here, we test the role of MLL3 in circadian organization in whole animals. Using mice expressing catalytically inactive MLL3, we show that MLL3 methyltransferase activity is in fact not required for circadian oscillations in vitro in a range of tissues, nor for the maintenance of circadian behavioral rhythms in vivo. In contrast to a previous report, loss of MLL3‐dependent methylation did not affect the global levels of H3K4 methylation in liver, indicating substantial compensation from other methyltransferases. Furthermore, we found little evidence of genomic repositioning of H3K4me3 marks. We did, however, observe repositioning of H3K4me1 from intronic regions to intergenic regions and gene promoters; however, there were no changes in H3K4me1 mark abundance around core circadian clock genes. Output functions of the circadian clock, such as control of inflammation, were largely intact in MLL3‐methyltransferase‐deficient mice, although some gene‐specific changes were observed, with sexually dimorphic loss of circadian regulation of specific cytokines. Taken together, these observations indicate that MLL3‐directed histone methylation is not essential for core circadian clock function; however, it may influence the inflammatory response.

**Significance**

It has been previously reported that there is an essential role for the histone methyltransferase MLL3 in maintaining circadian oscillations in cultured cells. We tested the role of MLL3 in vivo and in primary tissues but found that MLL3 did not affect the organization of the core circadian clock and had no functional impact on whole animal circadian behavior. However, in further analysis, we newly discover a role for MLL3 in conferring circadian control to components of the inflammatory response, doing so in a sexually dimorphic manner. As the MLL family of histone methyltransferases is being targeted by pharmaceuticals for cancer, it is important to understand how methyltransferases may be driving circadian rhythms in gene expression.

## 1 INTRODUCTION

The circadian clock drives the expression of thousands of genes in diverse tissues, conferring on them oscillating activity. The molecular clockwork consists of multiple transcriptional regulators which operate in a transcription–translation feedback loop (TTFL), with a period of approximately 24 h. Core clock components include BMAL1 and CLOCK, which form a heterodimer, capable of transactivating numerous target genes by binding to conserved E‐box elements. Among these targets are the PER and CRY genes, which form a transcriptional repressive complex to inhibit BMAL1/CLOCK transactivation. This completes the feedback loop. In addition, a second negative feedback loop arises from BMAL1/CLOCK transactivation of the NR1D1 orphan nuclear receptor. This binds to RORE elements on genomic DNA and recruits NCOR/HDAC3 repressor complexes to inhibit the expression of BMAL1. NR1D1 binds the same elements as RORa, a transactivator. In contrast to the high amplitude NR1D1 circadian oscillation, RORa expression is stable through time.

In addition to the well‐defined role of transcription factors in driving circadian oscillations in gene expression, there is evidence for dynamic changes in histone modifications associated with many circadian‐target genes, including Dbp, Per1, and Per2.^1^, ^2^, ^3^, ^4^ Regulation of histone modifications is likely to work in concert with the core clock TTFL, in order to confer specificity of transcriptomic rhythmicity to different tissues and cell types. To date, most research has focused on histone methylation and acetylation, and various marks, especially H3K4me1, H3K4me3, and H3K27ac, are found to show marked changes in abundance through circadian time series.^5^ Histone methyltransferases of the mixed lineage leukemia (MLL) family have emerged as likely effectors and, indeed, the expression of MLL1 and MLL3 may be clock controlled in specific tissues.^6^, ^7^ MLL3 was originally thought to induce H3K4me3 at the promoter regions of genes; however, more detailed investigations suggest that it mainly contributes to H3K4me1 at enhancer elements.^8^, ^9^, ^10^

MLL3 has been proposed as an epigenetic regulator required for the molecular circadian clock to function.^7^ Valekunja et al. reported MLL3 to be a clock‐controlled factor that directly or indirectly modulates over 100 epigenetically targeted circadian output genes in the mouse liver. They also showed almost complete loss of genome‐wide H3K4me3 deposition in MLL3 methyltransferase‐deficient mouse embryonic fibroblasts (MEFs). Furthermore, Valekunja et al. claimed that catalytic inactivation of MLL3 severely compromises the oscillation of core clock genes, including *Bmal1*, *Rev*‐*erbα*, and *Per2*, and concluded that lack of MLL3 methyltransferase activity controls both “core” and “output” clock genes.^7^

Here, we sought to extend the findings of Valekunja et al. to investigate the physiological role of MLL3‐dependent methylation in maintaining circadian organization, and temporal programming of tissue responses. Surprisingly, and in contrast to the previous report, we found that MLL3 methyltransferase activity was not required for core circadian function. Furthermore, we found almost no difference between genomewide H3K4me3 levels in MLL3 methyltransferase‐deficient mouse livers compared to wild‐type littermate controls. Similarly, there was only subtle repositioning of genomewide H3K4me1 deposition in liver between the two genotypes. Lastly, we examined the role of MLL3 in models of circadian output, namely, gating of immune function. Here, we found that MLL3 was required for subtle, and gene‐specific time of day variation of innate immunity. Taken together, we find that MLL3 is not required for the operation of the molecular circadian clock, but discover a subtle, yet distinct, role for MLL3 in coupling inflammatory signaling to the circadian clock. We cannot exclude a possible role for MLL3 in cells in which compensation for loss of MLL3 function is impaired, or defective, as may have been the case in the MEF cell studies reported before. However, a universal role for MLL3 in circadian function is certainly unlikely. 7

## 2 MATERIALS AND METHODS

### 2.1 Animals

All mice were routinely housed in 12:12 light/dark (L:D) cycles, unless otherwise stated, in light‐controlled chambers, with ad libitum access to food and water. Light exposure within the chambers was continuously monitored throughout experiments. All experiments were carried out in accordance with the Animals (Scientific Procedures) Act 1986 (UK). MLL3 Delta mice and MLL3^fl/fl^ mice were kindly provided by Professor Jae W. Lee. MLL3 Delta mice have a global in‐frame deletion of *Mll3* exons 57–58. Mice were bred as heterozygous pairs, producing mice homozygous for the Delta allele (MLL3 Delta mice), and animals homozygous for wild‐type MLL3 (wild‐type littermate controls). MLL3^fl/fl^ mice have exons 57–58 floxed, allowing recombination through cell‐type‐specific Cre expression. For experiments using conditional targeted mice, experimental animals were heterozygous for Cre expression, and controls were littermates (floxed/floxed) carrying no copies of the Cre. mPer2::luc mice were kindly provided by Professor Joe Takahashi.^11^ Locomotor activity was recorded in home cages using infrared beam‐break sensors, as previously described.^12^ Genotyping was performed on all animals, using previously published protocols.^13^, ^14^, ^15^

### 2.2 Bioluminescence recording

For ex vivo analyses of circadian rhythms, mice were bred onto an mPER2:luc genetic background, and tissues were harvested between 8 and 12 weeks of age. mPER2‐dependent bioluminescence was recorded from liver, lung, SCN slices, or cultured peritoneal macrophages (PECs), all maintained at 37°C using a Lumicycle (Actimetrics) as described previously.^16^ Circadian period was measured from the second peak post‐culture for two complete cycles. Two to three independent tissue samples were averaged for each animal. WDR5‐specific compound OICR9429 was purchased from Tocris and made up in DMSO. PECs were treated with OICR9429 at specified concentrations, with 0.1% DMSO final concentration in DMEM/F12 media.

### 2.3 Systemic LPS

Mice were challenged with intraperitoneal (IP) lipopolysaccharide (LPS; 1 mg/kg; isolated from *E. coli* 0127:B8, L4516, Sigma), at either ZT0 or ZT12, as previously described.^17^ Blood plasma was isolated 5 h after challenge. Cytokine analysis in plasma was performed by ELISA (R&D Systems) or Magpix assay (Luminex).

### 2.4 Aerosolized LPS

Mice were exposed to aerosolized LPS (2 mg/ml; isolated from *E. coli* 0127:B8, L4516, Sigma) or saline (0.9%) for 20 min, as previously described.^18^ Mice were euthanized 5 h after exposure. Bronchoalveolar lavage fluid was collected for cytokine secretion analysis by ELISA (R&D Systems). Lung tissue was collected for gene expression analysis by qPCR (SYBR Green, see table for primer sequences).

### 2.5 ChIP‐seq

Liver tissue was collected from MLL3 Delta mice and littermate wild‐type controls at ZT18. ChIP reactions were performed by Active Motif (Carlsbad, USA), using antibodies AB_2615075 and AB_2615077 for H3K4me1 and H3K4me3, respectively. The ChIP reactions also contained drosophila chromatin spike‐in for normalization of sequencing data. ChIP‐seq libraries were generated from the ChIP‐ and Input‐DNA using a custom Illumina library type on an automated system (Apollo 342, Wafergen Biosystems/Takara). ChIP‐Seq libraries were sequenced on Illumina NextSeq 500 as 75‐nt single end reads.

### 2.6 Data processing

Data were processed as previously published.^19^ QC on FastQ files was performed using FastQC v0.11.8.^20^ Trimmomatic v0.39^21^ was used to trim adapters and remove poor quality reads:

*java ‐jar trimmomatic‐0.39.jar SE ‐phred33 <FILENAME.fastq> ILLUMINACLIP:TruSeq3‐SE.fa:2:30:10 LEADING:3 TRAILING:3 SLIDINGWINDOW:4:15 MINLEN:36*.

Trimmed reads were aligned to the mm10 reference genome using Bowtie2 v2.4.1^22^:

*bowtie2 ‐p 6 ‐x mm10 ‐U <FILENAME.fastq> ‐S <OUTPUT_FILENAME.sam*).

SAMtools v1.10^23^ was used to produce BAM files from SAM files (*view*, *sort*, *index*, all with default settings).

Picard v2.22.9 was used to remove duplicate reads:

*java ‐jar picard.jar MarkDuplicates I=<FILENAME.bam> O=<OUTPUT_FILENMAE.bam> M=<METRICS_FILENAME.txt> REMOVE_DUPLICATES=true ASSUME_SORTED=true VALIDATION_STRINGENCY=LENIENT USE_JDK_DEFLATER=true*.

### 2.7 Peak calling

ChIP‐seq peaks were called in processed BAM files against processed control (input) files using MACS2 v2.2.7.1.^24^ Peaks were called using the following parameters:

*Macs2 callpeak ‐t <SAMPLENAMES.bam> c <INPUT.bam> ‐‐name <OUTPUT_FILENAME> ‐f BAM ‐g mm ‐‐keep‐dup=1 ‐q 0.01 ‐‐bdg ‐‐SPMR ‐‐verbose 0 > <OUTPUTFILENAME>*.

The narrowpeak setting was used to call peaks in the H3K4me3 data, while the broadpeak setting was used for H3K4me1 data (‐‐*broad*).

Peaks were counted using the respective commands:

*wc ‐l <FILENAME.narrowpeak> narrowPeak*.

*wc ‐l <FILENAME.broadpeak> broadPeak*.

BEDtools v2.29.2^25^ was used to find peaks which were common between datasets:

*bedtools intersect ‐u ‐a <FILENAME.broadPeak> ‐b FILENAME2.broadPeak > <OUTPUT_FILENAME.bed*>.

### 2.8 ChIP‐seq data visualization

deepTools v3.5.1^26^ was used to make heat maps of signal intensity, using the commands *computeMatrix* and *plotHeatmap*. Integrative Genomics Viewer v2.9.4^27^ was used to produce visualizations of ChIP‐seq data tracks.

### 2.9 Statistics

Statistical tests and sample numbers are specified in figure legends where appropriate. Unless otherwise stated, statistical significance was tested using Mann–Whitney U test. Statistical tests were conducted in GraphPad Prism. Throughout, * denotes *p* < .05, ** denotes *p* < .01, *** denotes *p* < .001 and plots were produced in GraphPad Prism, except for ChIP‐seq data, which is described above.

## 3 RESULTS

### 3.1 MLL3 Delta mice show normal behavioral circadian rhythms

MLL3 functions as part of a multiprotein complex known as ASCOM, which also contains RbBP5, ASH2L, WDR5, UTX, and other additional proteins.^28^ ASCOM may contain either MLL3 or MLL4 as the active H3K4 methyltransferase, while UTX demethylates H3K27. ASCOM may also recruit CBP/p300 to acetylate H3K27, further facilitating gene transcription.^13^, ^29^ To explore the biology of MLL3‐mediated methylation specifically, we used a strain of mice (MLL3 Delta) which lacked the catalytic domain of the MLL3 enzyme due to the in‐frame deletion of exons 57–58.^13^ This mutation renders MLL3 unable to catalyze methylation of H3K4, while preserving structural functions. The previous reports of a striking circadian role for MLL3 were defined using isolated MEF cells from these same MLL3 Delta mice in culture.^7^ Here, we bred MLL3 Delta mice on a mixed C57Bl6/129sv genetic background, since this mutation is embryo‐lethal on a pure C57Bl6 background (Figure S1A).^8^ Using this breeding strategy, we were able to generate viable mice, homozygous for the MLL3 catalytic domain mutation (henceforth referred to as MLL3 Delta mice), and wild‐type littermate controls. We observed fewer live‐born MLL3 Delta animals, in line with previous observations,^8^ suggesting that MLL3 catalytic deficiency imposes a toll in animal development (Figure S1B). No changes were observed in transcriptional expression of the related methyltransferases MLL1, 2, and 4 (Figure S1C). We were also able to confirm that MLL3 expression levels were unaffected, despite the expected in‐frame deletion of exons 57 and 58 (Figure S1C).

**FIGURE 1:**
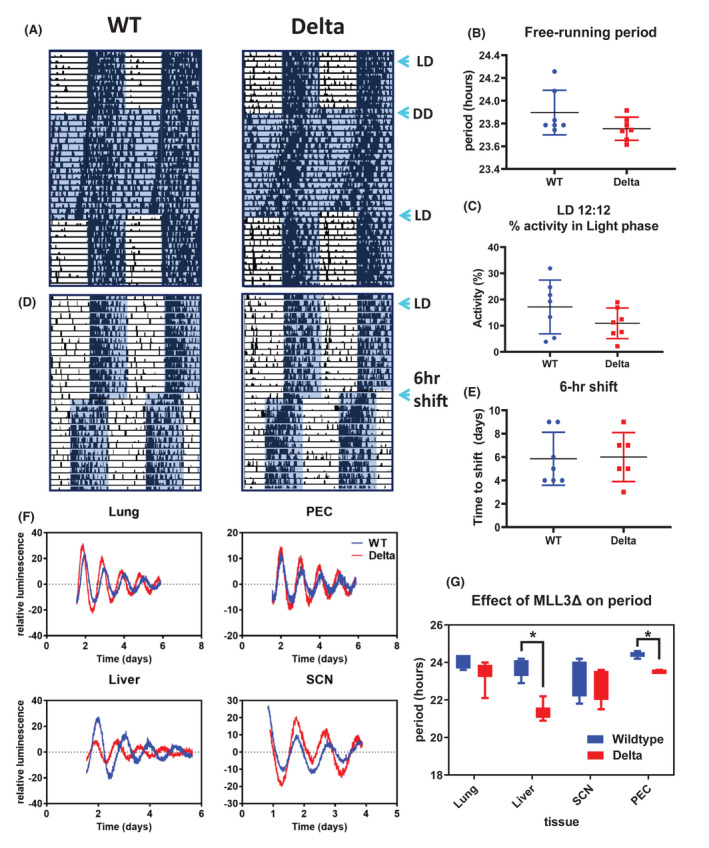
Circadian rhythms persist in the absence of MLL3 methyltransferase function. (A) Mice expressing global MLL3 Delta and littermate wild‐type controls were placed in light control cabinets in 12:12 light dark (L:D) cycles for 2 weeks to acclimatize. Mice were then exposed to constant dark for 2 weeks and allowed to “free‐run,” before returning to a 12:12 L:D exposure. Mouse activity was monitored using beam‐break technology. (B) The free‐running period was calculated during 2 weeks of constant darkness. (C) The percentage of activity in the light phase of each animal was quantified during 1 week in 12:12 L:D conditions. (D) After another 2‐week acclimatization period to 12:12 L:D conditions, mice were exposed to a 6‐h phase advance light shift. (E) The time‐to‐shift and re‐entrain with the phase advance light pattern were quantified. (F) Lung, liver, suprachiasmatic nuclei (SCN) and peritoneal macrophage (PEC) tissue explants from MLL3 Delta mice and wild‐type littermate controls on a Per2::luc genetic background were placed in a lumicycle to observe circadian oscillations in PER2 expression. (G) The circadian period in each of the tissues was quantified and compared between the two genotypes (*n* = 3–5).

The mice were left to free‐run in constant darkness (Figure 1A). Free‐running period was slightly but nonsignificantly shorter in MLL3 Delta mice compared to littermate controls and less than 24 h for both genotypes (Figure 1B), consistent with previously published observations of mice on a C57Bl6 genetic background. In further analyses, we measured activity during the light and dark phases (Figure 1C) and found no genotype differences. We next tested phase shifting responses, and exposed mice to a 6‐hour phase advance (Figure 1D). There was no significant difference between the MLL3 Delta mice and the WT littermate controls in the time taken to reset (Figure 1E). These findings were strikingly different from predictions based on the in vitro analysis of MLL3 circadian function.^7^

As previous reports had detected a major role for MLL3 in isolated cells in culture, we considered that the minimal effects observed in vivo may result from masking phenomena arising from a dominant central pacemaker. Therefore, we crossed the MLL3 delta mice onto the Per2::Luc genetic background. We isolated four tissue types, namely liver, lung, suprachiasmatic nucleus (SCN), and peritoneal macrophages (PEC), and monitored these cells, and tissue explants in culture through time, monitoring light production in a lumicycle (Figure 1F,G). In marked contrast to the earlier report,^7^ all isolated tissues showed strong, sustained in vitro circadian oscillations, allowing detailed analysis of waveforms. SCN and lung explants showed no significant genotype difference. In contrast, both liver and PECs had a significantly shorter circadian period in MLL3‐delta‐derived tissues (Figure 1G).

### 3.2 ChIP‐seq reveals no global change in H3K4me3 levels in liver

**FIGURE 2:**
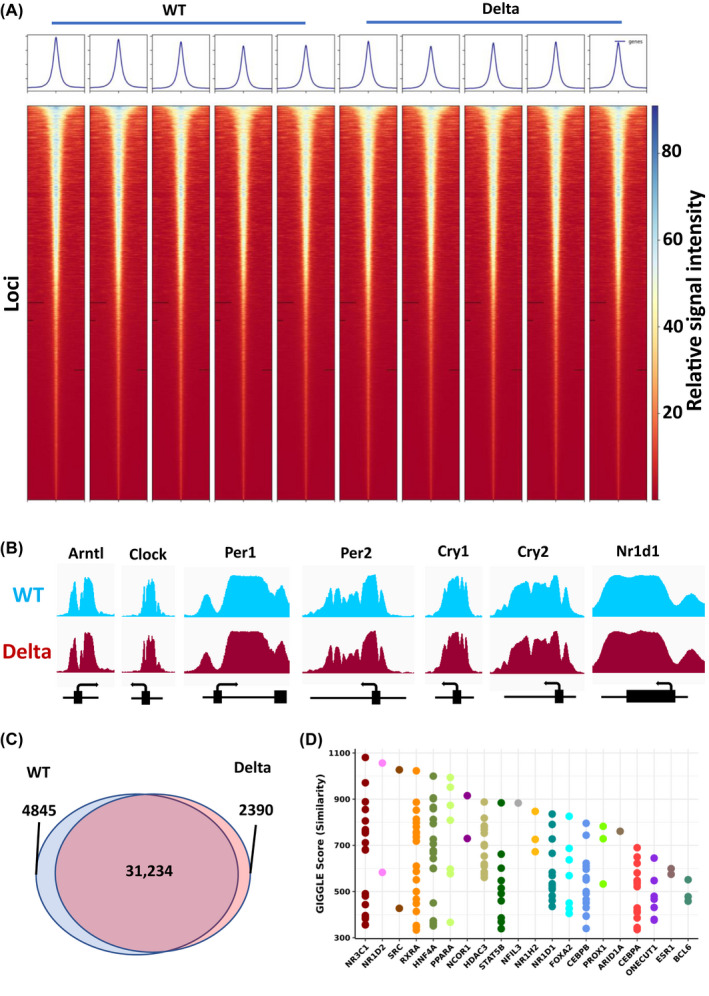
H3K4me3 ChIP‐seq analysis of liver tissue from MLL3 Delta mice and WT littermate controls. (A) Individual heat maps of MACS2‐called H3K4me3 peak intensities from *n* = 5 Delta and *n* = 5 control WT mice were plotted. 5 kb is shown either side of the peak summit. (B) MACS2‐derived Bedgraph files for all samples within a group were converted to TDF files to view ChIP‐seq gene tracks in IGV. Gene tracks across the promoter and TSS of core clock genes are shown for the WT and MLL3 Delta genotypes. (C) MACS2 was also used to call peaks from the BAM files. The Venn diagram shows the total number of overlapping and nonoverlapping peaks called within the WT and Delta samples. (D) Peaks which were found to be unique to the WT condition were submitted for Giggle score analysis to assess similarity with other published ChIP‐seq factor‐binding datasets.

To investigate further possible mechanisms underpinning the altered circadian phenotype seen in hepatic tissue, we performed ChIP‐Seq for the H3K4me3 mark in liver from MLL3 Delta animals and wild‐type littermate controls. We selected Zeitgeber‐time 18 (ZT18), as this time point had previously been proposed as the peak for H3K4me3 levels and genomic MLL3 association.^5^, ^7^ Strikingly, we observed remarkably few differences between MLL3 Delta animals and wild‐type littermate controls. In contrast to previous findings,^7^ we identified no impact of MLL3 Delta on global H3K4me3 levels (Figure 2A). We further noted there was no observable difference in H3K4me3 levels at the promoter of any core clock gene, in agreement with the intact circadian oscillations observed in mouse behavior and isolated tissues (Figure 2B). In total, 36 079 H3K4me3 peaks were identified in wild‐type mice, of which 31 234 (87%) were also identified in the MLL3 Delta mice, highlighting the remarkable similarity between the genotypes (Figure 2C). Indeed, principal component analysis did not separate the animals by genotype, only by sex (Figure S2B).

### 3.3 H3K4me3 does not explain changes in period

As we observed a significant shortening of period in the MLL3 Delta liver explants, we looked for a potential mechanism involving MLL3 catalytic activity in the H3K4me3 ChIP‐seq data. To investigate this, we identified the H3K4me3 peaks which were unique to the wild types or MLL3 Deltas, and also located at a canonical gene promoter, and compared these genes with those listed in the “Circadian rhythm genes” GO term (Figure S2A). Only two “circadian genes” which lost promoter H3K4me3 peaks were identified, *Drd1* and *Slc6a4*, and one “circadian gene” gained a H3K4me3 peak in the MLL3 Deltas, *Nrg1*. None of these three genes are known to have a role in determining core clock period nor are they typically well expressed in liver. Therefore, the altered period length seen in MLL3 Delta liver explants (Figure 1F) is not likely to be due to altered H3K4me3 deposition affecting promoter function of the core clock.

To take an unbiased view of the subtle changes in H3K4me3 deposition between the two genotypes, we performed GIGGLE analysis on the loci of the unique wild‐type peaks.^30^ The wild‐type unique peaks shared genomic loci with well‐characterized MLL3 recruiting transcription factors including the glucocorticoid receptor (NR3C1), and interestingly, the key circadian output regulator REVERBb (NR1D2) (Figure 2D). In addition, there was enrichment for other circadian, or circadian output factors, including PPARA, STAT5B, NFIL3, REVERBa (NR1D1), and NCOR1.^31^, ^32^, ^33^, ^34^, ^35^, ^36^ These overlaps hint that MLL3 enzymatic activity may play a role in tuning circadian output functions, without contributing to the core circadian oscillator.

### 3.4 Minor repositioning of H3K4me1 in MLL3 Delta liver

**FIGURE 3:**
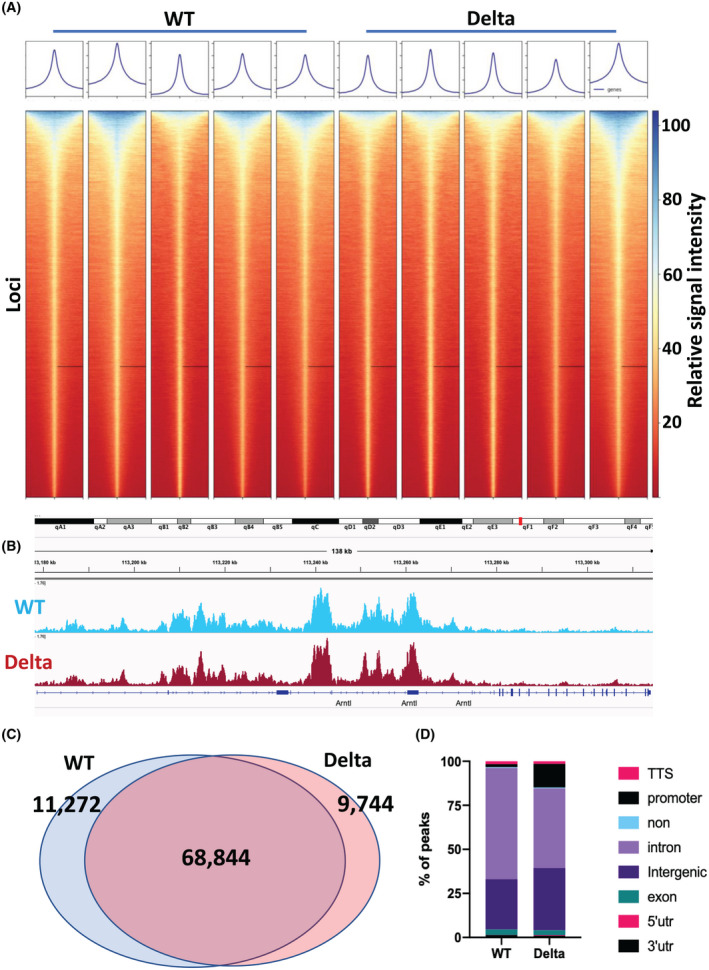
H3K4me1 ChIP‐seq analysis of liver tissue from MLL3 Delta mice and WT littermate controls. (A) Individual heat maps of MACS2‐called H3K4me1 peak intensities from *n* = 5 Delta and *n* = 5 control WT mice were plotted. 5 kb is shown in either side of the peak summit. (B) H3K4me1 gene tracks for WT and Delta conditions are shown across the *Bmal1* (*Arntl*) locus. (C) Venn diagram showing the overlap of MACS‐called broad peaks in the WT and Delta conditions. (D) The relative proportion of peaks across different types of genomic regions was quantified.

Recent studies have shown that MLL3 exhibits higher activity depositing monomethyl than trimethyl marks at H3K4.^9^, ^37^, ^38^, ^39^ Mono‐methylation of H3K4 denotes enhancers and deposition of this mark has also been linked to circadian transcription.^5^ To pursue the molecular basis of the altered circadian period length seen in isolated liver tissue, we then moved to profile changes in H3K4me1 in livers from the two genotypes (Figure 3). Similar to the H3K4me3 dataset, there was no gross change in the global levels of H3K4me1 marks across the genome (Figure 3A). Similarly, no differences were found in H3K4me1 deposition across circadian genes (Figure 3B). However, some subtle differences were identified between the two genotypes. In total, 80 116 H3K4me1 peaks were identified in the wild type, of which 68 844 were also found in the MLL3 Delta mice (Figure 3C). Furthermore, 9744 peaks were found only in the MLL3 Deltas. Interestingly, there appeared to be a subtle, but distinct, redistribution of peaks from introns to intergenic and promoter regions in the MLL3 Delta mice (Figure 3D). We examined reads arising from the MLL3 gene locus, and, as a useful additional control for the efficiency of gene recombination, we saw a complete absence of reads from the targeted MLL3 exons 57 and 58 (Figure S3A).

Analysis of the genotype unique peaks by GIGGLE revealed a surprising overlap with the variant histone H2AZ at gained peaks in the MLL3 delta, but we did not see enrichment for circadian transcription factors in either condition (Figure S3B,C).

### 3.5 Macrophage‐specific deletion of MLL3 catalytic region does not affect circadian period

**FIGURE 4:**
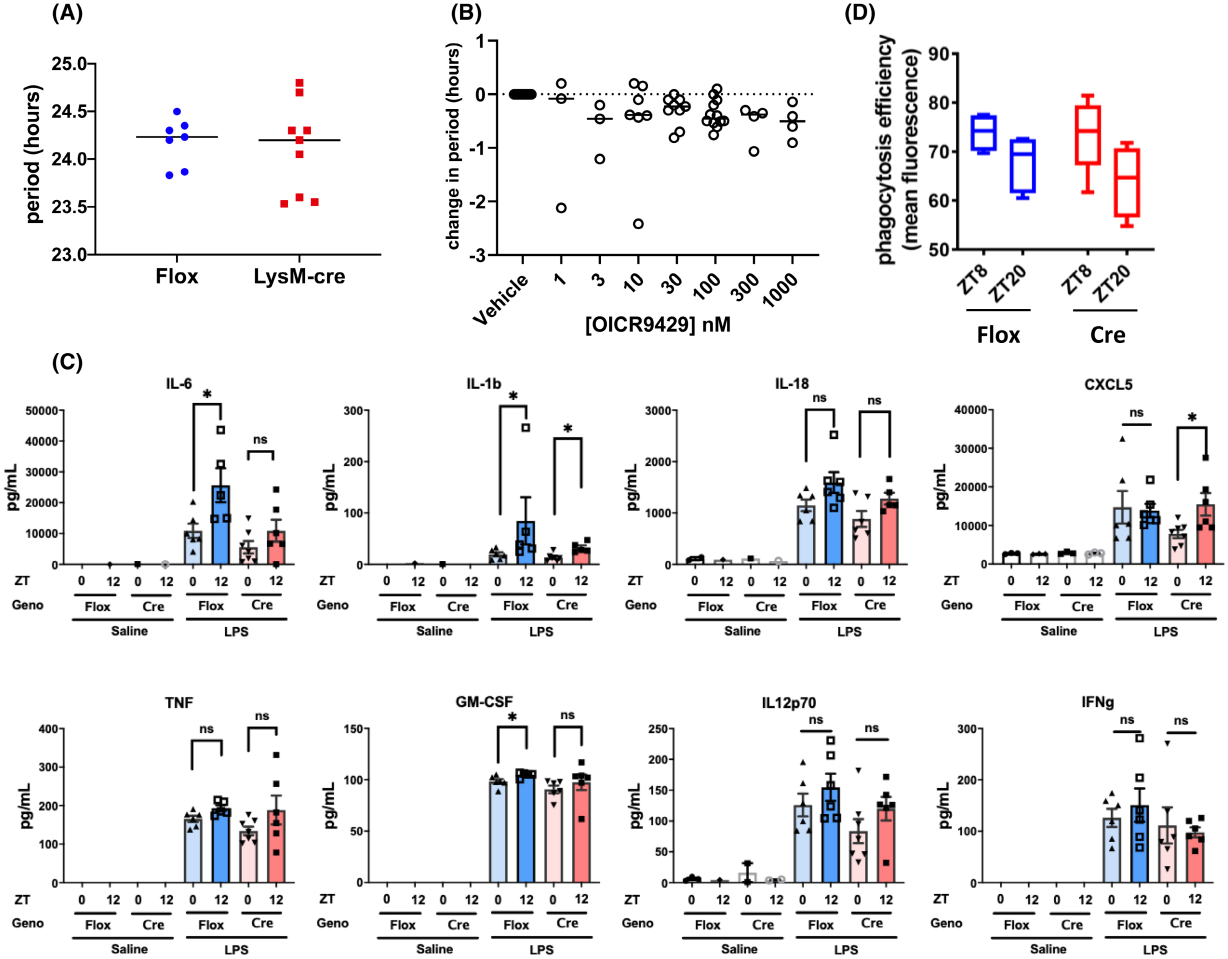
The role of MLL3 in the circadian function of macrophages. (A) PECs were isolated from MLL3^Delta flox/Delta flox^.LysMcre^het^ mice and MLL3^Delta flox/Delta flox^ littermate controls, and placed into a lumicycle. Circadian oscillations in Per2::luc were measured for 3 days and period was calculated. Each data point represents the average of three technical replicates from an individual animal. (B) PECs were isolated from Per2::luc mice and plated in 96‐well plates before treatment with varying concentrations of selective WDR5 inhibitor OICR9429 or vehicle control (0.1% DMSO). Oscillations in Per2::luc were measured using a Clariostar plate reader and period was calculated from 24 to 72 h after plating. (C) MLL3^Delta flox/Delta flox^ LysMcre^het^ mice and MLL3^Delta flox/Delta flox^ littermate controls were exposed to I.P. LPS at ZT0 or ZT12. Six hours after exposure cytokine levels in blood plasma were measured by ELISA or Magpix. (D) PECs were isolated from MLL3^Delta flox/Delta flox^ LysMcre^het^ mice and MLL3^Delta flox/Delta flox^ littermate controls at ZT8 or ZT20 and plated with pHrodo‐Staph. Aureus bioparticles. Efficiency of phagocytosis was measured by flow cytometry (*n* = 4–6).

The isolated SCN appeared to show no genotype‐dependent circadian phenotype, and its operation in vivo may be masking the role of MLL3 on clock function in peripheral cells. Since macrophage cells did show significant period shortening in MLL3 Delta mice, we explored this further and targeted MLL3 deletion to specific cells using cell‐type‐specific cre recombinase (Lysozyme Cre) crossed to a floxed allele of MLL3 to target macrophage cells. Surprisingly, and in contrast to macrophages recovered from the global MLL3 Delta animals, the peritoneal macrophages showed no genotype differences (Figure 4A).

While MLL3 drives the active chromatin H3K4 methyl marks, another enzyme in the COMPASS complex, UTX, removes H3K27me3 marks. We therefore speculated that another histone methyltransferase, EZH2, which deposits H3K27me3, may have a role in circadian function in macrophages. EZH2 has been reported to affect circadian function, and impacts on innate immunity, and macrophage function.^40^, ^41^ Therefore, we analyzed isolated peritoneal macrophages from the *CX3CR1*‐*cre*.*EZH2^f^* ^/^ *^f^* animals, compared to the cre‐negative littermates (Figure S4A). Here again, we did not detect any circadian phenotype, despite the earlier report of a strong circadian phenotype in zebrafish.^41^ We also analyzed liver tissue from a liver‐specific EZH2 knockout mouse, on the basis that isolated liver tissue from the MLL3 disrupted animals showed a circadian phenotype, using a tamoxifen‐directed deletion strategy to reduce confounding by genetic compensation. However, again, no circadian phenotype was seen (Figure S4B). Therefore, EZH2 did not contribute to circadian organization in our models.

A degree of functional redundancy between MLL3 and MLL4 within the COMPASS complex has been previously reported.^8^, ^13^, ^42^ It is possible that this functional redundancy may mask an important role for COMPASS‐directed H3K4 methylation in circadian cycling. Therefore, we tested an inhibitor of WDR5, which is the common structural platform of the COMPASS complex facilitating the interaction of the methyltransferase enzyme with the histone substrate.^43^ However, there was no observed dose‐dependent change in circadian period in isolated macrophages treated with WDR5 antagonist, OICR9429 (Figure 4B).

Taken together, these observations may indicate that the differences in period observed in the global MLL3 Delta mice, may not be directly due to changes in MLL3‐dependent histone methylation, but possibly reflect subtle genetic background effects.

### 3.6 Time of day gating of inflammatory responses is not dependent on MLL3

Circadian gating mechanisms are important in immunity and host defense (Scheierman et al. review). Since our analysis of the MLL3 disruption revealed changes in relation to binding sites of a number of inflammation regulatory transcription factors with reported roles in mediating circadian control, including NR3C1, NR1D1, NR1D2, and NFIL3 (Figure 2D), we next investigated the role of MLL3 in conferring temporal control to inflammation. For this, we used *MLL3^f^* ^/^ *^f^*.*LysMcre* mice and *MLL3^f^* ^/^ *^f^* littermate controls. Animals were exposed to lipopolysaccharide (LPS), via an intraperitoneal route. We have previously characterized this model and shown enhanced inflammatory responses at ZT12 relative to ZT0, which is dependent on macrophages.^17^

Analysis of circulating cytokine concentrations revealed a subtle impact with some (IL‐6) showing a loss of temporal regulation in the absence of MLL3, but others (e.g., IL‐1b) showed preserved variation (Figure 4C). Other cytokines, including TNF, GM‐CSF, IL12p70, and INFg showed no time of day gating, and no significant differences between genotypes (Figure 4C). As we have recently discovered a strong circadian clock regulation of macrophage phagocytosis, we also measured this by time of day in the two genotypes (Figure 4D). Here, we again saw time of day variation in phagocytosis efficiency as previously reported, but no genotype effect.

**FIGURE 5:**
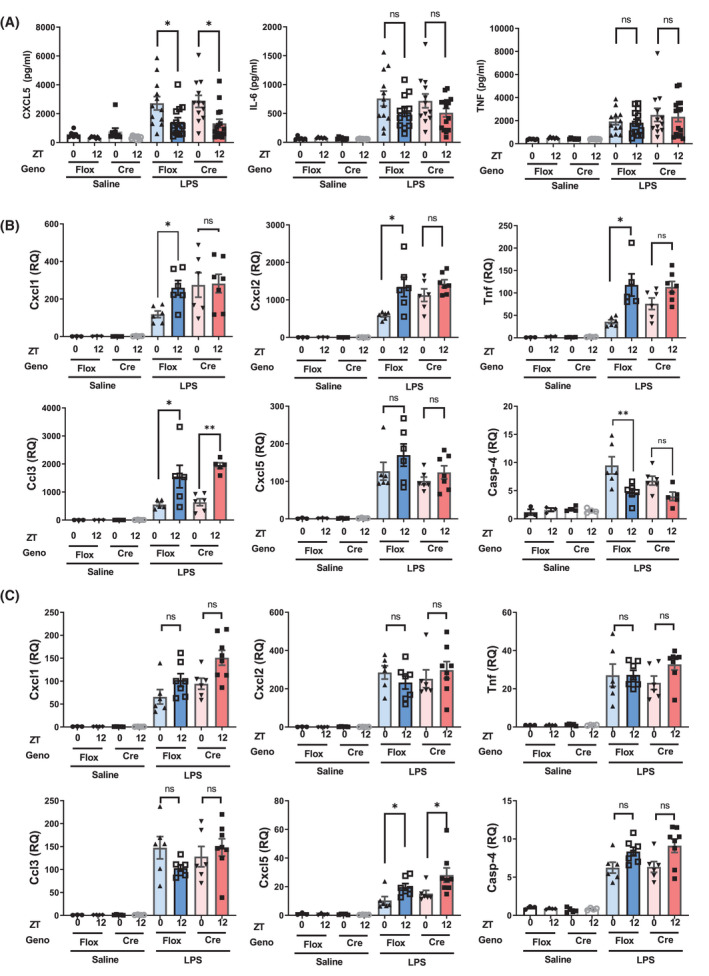
The role of MLL3 in the circadian gating of the airway inflammatory response. MLL3^Delta flox/Delta flox^ CCSPiCre^het^ mice and MLL3^Delta flox/Delta flox^ littermate controls were exposed to aerosolized LPS at ZT0 or ZT12. Five hours after exposure bronchoalveolar lavage fluid (BALF) and lung tissues were harvested. (A) Cytokine levels were quantified in the BALF by ELISA. mRNA levels of various genes associated with the inflammatory response were quantified by qPCR. (B) The quantified levels in male mice. (C) The quantified levels in female mice.

Because pulmonary inflammatory responses are strongly circadian, we next moved to an aerosolized LPS model to target the lung directly. Here, we have previously characterized enhanced inflammation at ZT0 relative to ZT12, which is gated by the circadian clockwork of lung epithelial cells.^18^ Therefore, we targeted the MLL3 Delta mutation specifically to lung epithelial cells using a CCSP‐iCre driver (*MLL3^f^* ^/^ *^f^*.*CCSPiCre*). We first profiled the response to nebulized lipopolysaccharide administered at ZT0, or ZT12 in the *MLL3^f^* ^/^ *^f^*.*CCSP^icre^* mice and *MLL3^f^* ^/^ *^f^* littermate controls by assaying bronchoalveolar lavage fluid (Figure 5A). Here, we saw no genotype effect on responses to bronchoalveolar lavage fluid concentration of CXCL5, IL6, or TNFa, characteristic cytokine/chemokine‐responsive proteins (Figure 5A). The previously reported time of day variation in CXCL5 concentration was seen in both genotypes.^18^ Furthermore, the absence of MLL3 catalytic activity did not affect the circadian regulation of *Rev*‐*erbα* by time of day, or by LPS (Figure S5A).

In a further analysis, we measured inflammatory mediator mRNA expression in lung tissue from the same animals. Here, male and female mice showed significant differences in response, and so were analyzed separately (Figure 5B,C). We observed a surprising difference between the two genotypes, in males, with time of day variation in CXCL1, CXCL2, TNFa, and Caspase 4 all being lost in the absence of MLL3 (Figure 5B). The peak inflammatory chemokine expression in lung tissue is in anti‐phase to that of the mature protein secreted into the airway, reflecting further complexity in the circadian regulation of integrated lung immune responses. This may in part be due to circadian regulation of the *Tlr4* receptor in the lung tissue, which was increased at ZT12 in both genotypes (Figure S5B). The loss of circadian gating did not extend to all measured transcripts, with CCL3 showing a peak at ZT12 in both genotypes (Figure 5B). Intriguingly, we did not see a time of day or genotype difference in lung CXCL5 gene expression in males (Figure 5B). In females, the time of day variation in gene response was confined to CXCL5, and here, we saw no impact of MLL3 loss (Figure 5C). This suggests that MLL3 is driving different biology in males and females. As MLL3 is a transcriptional co‐activator, driving methylation of H3K4, and thereby promoting active promoters and enhancers, it is further surprising that in the absence of MLL3, the dawn nadir of chemokine gene expression was lost. This suggests a gain in gene expression, a change perhaps best seen in CXCL1.

## 4 DISCUSSION

In the present study, we set out to investigate the interaction between the histone methyltransferase MLL3 and the core circadian clock. The core clock TTFL controls the circadian oscillation of thousands of individual transcripts and, importantly, is able to do so in a tissue‐specific manner. Furthermore, the core clock gates specific cellular functions by time of day, including the inflammatory response. In order to facilitate such diverse yet fine‐tuned genomic control, the core clock must coordinate with histone modifiers, such as MLL3. We investigated the effect of MLL3 Delta in mouse behavioral rhythms, but found no differences between animals lacking MLL3 methyltransferase activity, and their wild‐type littermates. Similarly, isolated mouse tissues SCN, lung, liver, and isolated macrophages exhibited strong circadian oscillations in the absence of MLL3 catalytic activity, in stark contrast to the previously published observations in MEFs.^7^ However, we did observe tissue‐specific changes in period in liver and peritoneal macrophages in mice with global MLL3 Delta mutation. To investigate the apparent disconnect between our observations in mature tissues, and those previously published in MEFs, we performed H3K4me1 and H3K4me3 ChIP‐seq in liver tissue from MLL3 Delta mice and littermate wild‐type controls. We found surprisingly subtle changes in genomic histone methylation, explaining the lack of circadian phenotype and implying compensation from other methyltransferases.

Given the strong effect of MLL3 Delta on development, we were surprised to find only subtle effects on the circadian clock. In the surviving MLL3 Delta homozygotes, no effect on circadian behavioral parameters was observed, and in corroboration, oscillations in isolated SCN were equivalent between Delta mice and wild‐type littermate controls. Intriguingly, we observed a significant shortening of circadian period in other isolated tissues, specifically liver and peritoneal macrophages. This indicated that there may be some subtle interactions between MLL3‐dependent methylation and core clock function in a tissue‐specific manner; however, it is difficult to reconcile these observations with the stark and complete loss of oscillation reported in MEFs.^7^

In order to investigate the difference between our observations and those in MEFs, we performed ChIP‐seq. Circadian oscillations in global H3K4me3 peaking at CT18 have previously been reported.^5^, ^7^ We therefore harvested liver tissue from MLL3 Delta mice and wild‐type littermate controls at ZT18 and performed H3K4me3 ChIP‐seq. Again, in stark contrast to the findings of Valekunja et al., we found no difference in the overall global levels of H3K4me3 deposition between the two genotypes, and only subtle repositioning of the mark in the Delta condition. Furthermore, we found no differences in the H3K4me3 deposition across the promoters of the core clock genes, explaining why a fully functional circadian clock was observed in MLL3 Delta liver tissue. Our observations are in line with other reports that find little impact of MLL3 catalytic inactivation on gene expression.^38^, ^44^, ^45^ This may be due to functional redundancy with MLL4^8^, ^42^

Detailed reports have found that MLL3 has considerably stronger activity depositing H3K4me1, and that H3K4me3 is mostly deposited by other methyltransferases, such as MLL1.^6^ H3K4me1 denotes enhancers, which modulate the expression of genes from a range of thousands to millions of base pairs away. For this reason, it is difficult to assign changes in enhancer H3K4me1 deposition to changes in gene expression, and examining the H3K4me1 levels proximal to genes of interest is not necessarily informative. Regardless, little difference in H3K4me1 levels was found in the loci of core clock genes. Furthermore, the global levels of H3K4me1 deposition were comparable between the two genotypes and, as with H3K4me3, only subtle repositioning of H3K4me1 peaks was observed. Interestingly, globally, there was a redistribution of H3K4me1 peaks from intronic regions to intergenic and promoter regions in the MLL3 Delta liver tissue compared to the wild‐type littermate controls (Figure 3D). This may be due to a compensation of other histone methyltransferases with a selectivity for different genomic regions/substrates to that of MLL3. Indeed, GIGGLE score analysis identified an overlap of MLL3 Delta‐specific H3K4me1 peaks with previously published H2A.Z ChIP‐seq peak locations, which may indicate that in the absence of MLL3 catalytic activity, the COMPASS complex is being drawn to more active chromatin.

We next examined whether MLL3 methyltransferase activity may confer time of day control to known circadian‐regulated processes. We chose to examine the inflammatory response, which we have previously extensively characterized, is strongly circadian.^17^, ^18^, ^33^, ^46^ We examined two models of TLR4‐driven inflammation, the first a systemic response to IP injection of LPS, which we have previously shown is gated by the macrophage cell clock, and secondly aerosolized LPS, which shows a circadian response that is gated by epithelial cell clocks. Interestingly, the enhancement of inflammation in these two models is in anti‐phase: enhanced inflammatory response in the systemic model is observed at ZT12, whereas enhanced inflammatory response in the aerosolized model is observed at ZT0, indicating different circadian mechanisms.

One of the key cytokines driving the inflammatory response in the systemic model (IP LPS) is IL‐6. Time of day gating of IL‐6 was lost in the MLL3^flox^.LysMcre mice; however, only subtle differences were seen in the circulating concentrations of other cytokines. By contrast, the circadian gating of the airway response to aerosolized LPS is driven by CXCL5 secretion.^18^ This time of day gating was maintained in MLL3^flox^.CCSPiCre mice indicating a specificity of MLL3 action in conferring circadian control. In the aerosolized LPS model, we went on to look at the mRNA induction in the lung tissue. Surprisingly, we found that the circadian peak of many inflammatory genes was in anti‐phase to the peak concentration of cytokines secreted into the airway lumen. In the lung tissue, we found a sexually dimorphic inflammatory response, in line with previous publications.^47^, ^48^ Furthermore, we found a sex‐specific role for MLL3 in the gating of the inflammatory response, for example, in the case of *Cxcl1* and *Tnf*. It is of note that in some cases where significance of circadian differences in cytokine expression was lost, a residual trend in the same valence was still observed.

In summary, we find that MLL3 is not required for circadian cycling of core clock genes, in strong contrast to previous reports. We do, however, find subtle, sex‐specific effects of MLL3 on H3K4me1 distribution across the genome, and on circadian gating of the inflammatory response in peripheral tissues. Further investigations into the interaction of histone‐modifying complexes and the circadian clock are warranted, and may lead to better understanding of the inflammatory response in relation to time of day specificity and sexual dimorphism.

## AUTHOR CONTRIBUTIONS

Matthew Baxter, Andrew S. I. Loudon, and David W. Ray designed the study. Matthew Baxter, Toryn Poolman, Peter Cunningham, Louise Hunter, Maria Voronkov, Gareth B. Kitchen, Laurence Goosey, Nicola Begley, Danielle Kay, Abby Hespe, and Robert Maidstone performed data collection and/or bioinformatic analysis. Matthew Baxter and David W. Ray wrote the manuscript. Andrew S. I. Loudon, Peter Cunningham, Louise Hunter, Robert Maidstone, and Maria Voronkov supported manuscript preparation.

## DISCLOSURES

The authors declare no competing interests.

## Supporting information

Fig S1‐5

## Data Availability

ChIP‐seq data have been uploaded to ArrayExpress. Accession number E‐MTAB‐1120.
