# Supplementary material for: Circadian clock function does not require the histone methyltransferase MLL3
Source: FASEB J. 2022 Jun 15;36(7):e22356. doi: 10.1096/fj.202200368R (PMC9328146; doi:10.1096/fj.202200368R)
Supplement: Supplementary file 1 — Fig S1‐5 [file FSB2-36-0-s001.pdf]

Figure 1 supplement

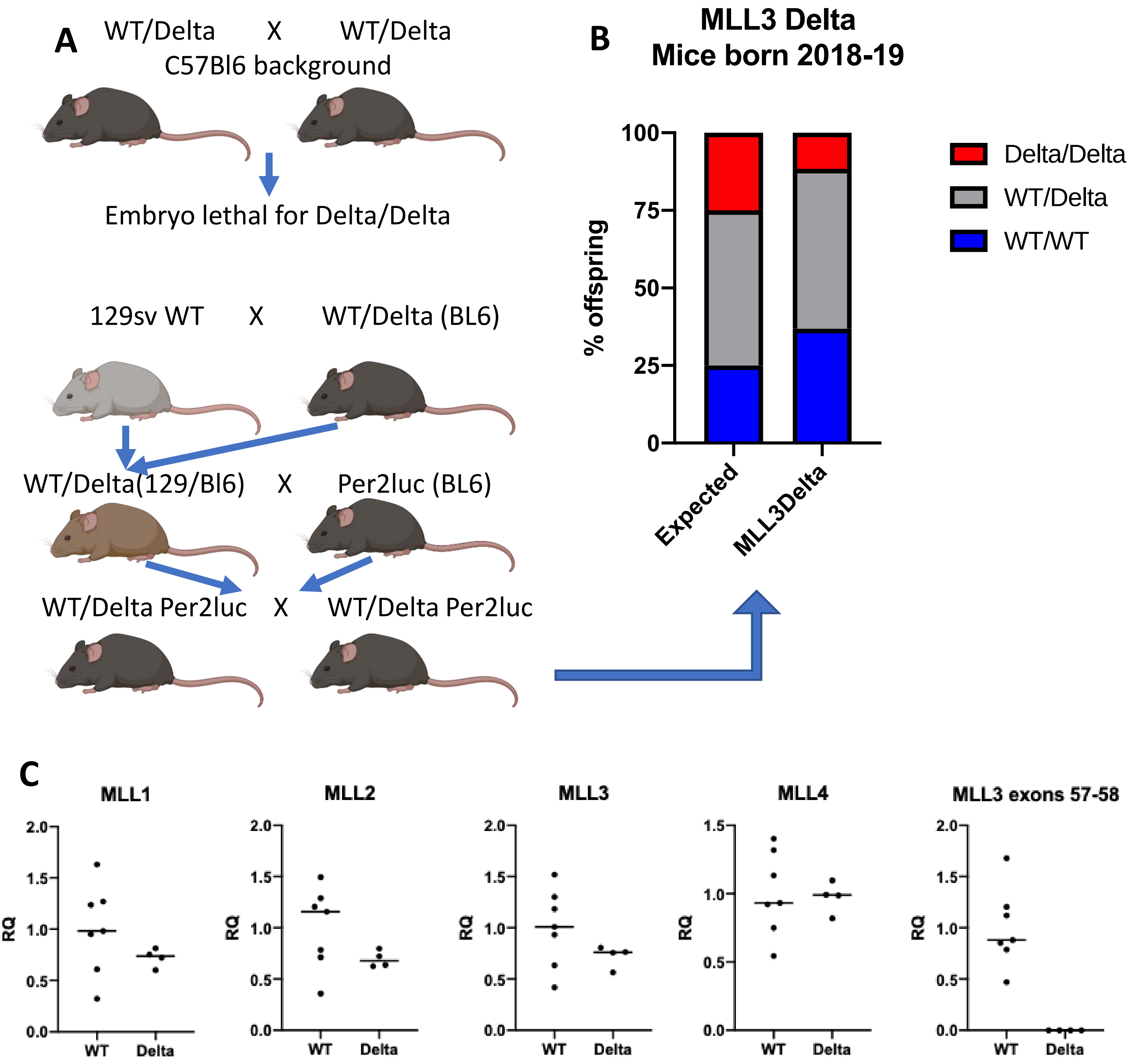

A. Breeding scheme to show the development of MLL3 Delta and wild-type control mice on a mixed C57Bl6/129s background, expressing Per2::luc. B. quantification of viable mice born from MLL3 Delta heterozygous crosses on C57Bl6/129s background. The number of mice which were homozygous, heterozygous and wild-type for the MLL3 Delta mutation is shown, alongside the expected Mendelian ratio. C. mRNA expression analysis of MLL3 alleles and other MLL family members which may form part of the COMPASS complex, from MLL3 Delta animals and wild-type controls.

Figure 2 supplement

A

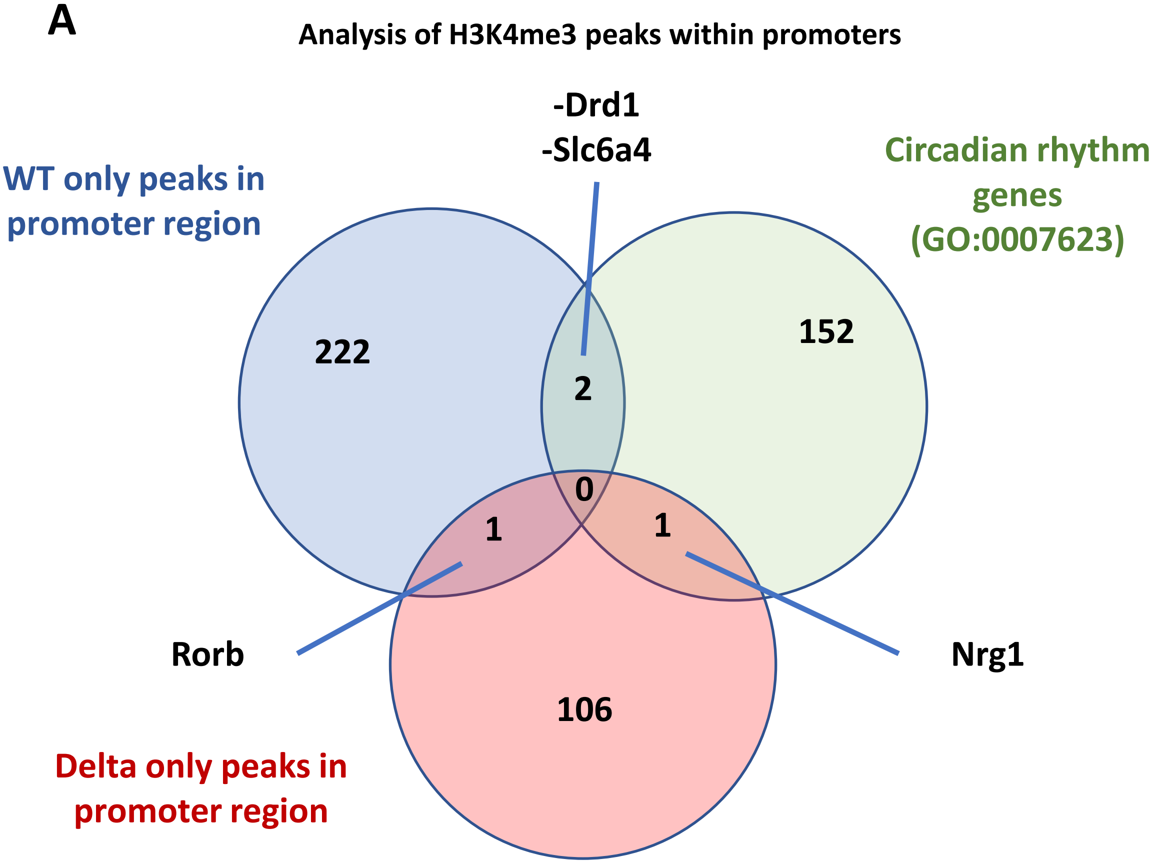

B

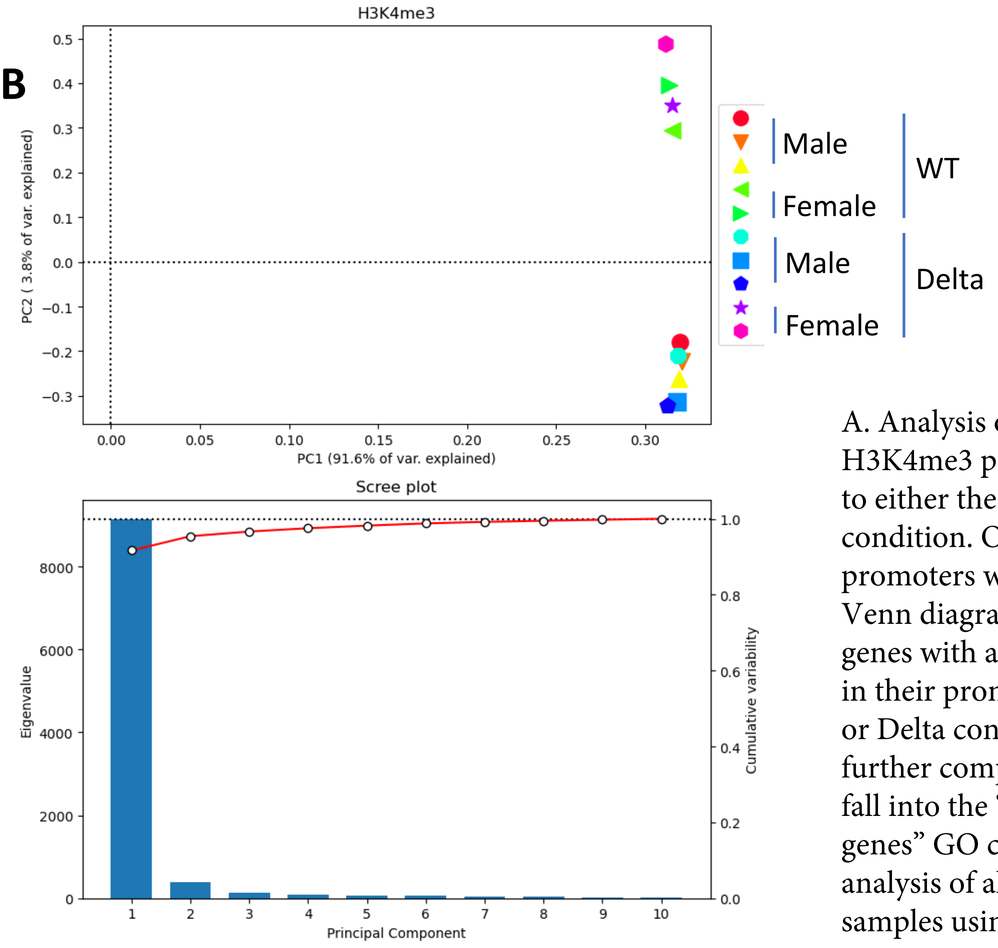

A. Analysis of positioning of H3K4me3 peaks which were unique to either the WT or Delta condition. Only peaks within gene promoters were analysed. The Venn diagram shows the number of genes with a unique H3K4me3 peak in their promoter in either the WT or Delta condition. These genes are further compared with genes that fall into the “Circadian rhythm genes” GO category. B. PCA analysis of all H3K4me3 ChIP-seq samples using Deeptools.

Figure 3 Supplement

A

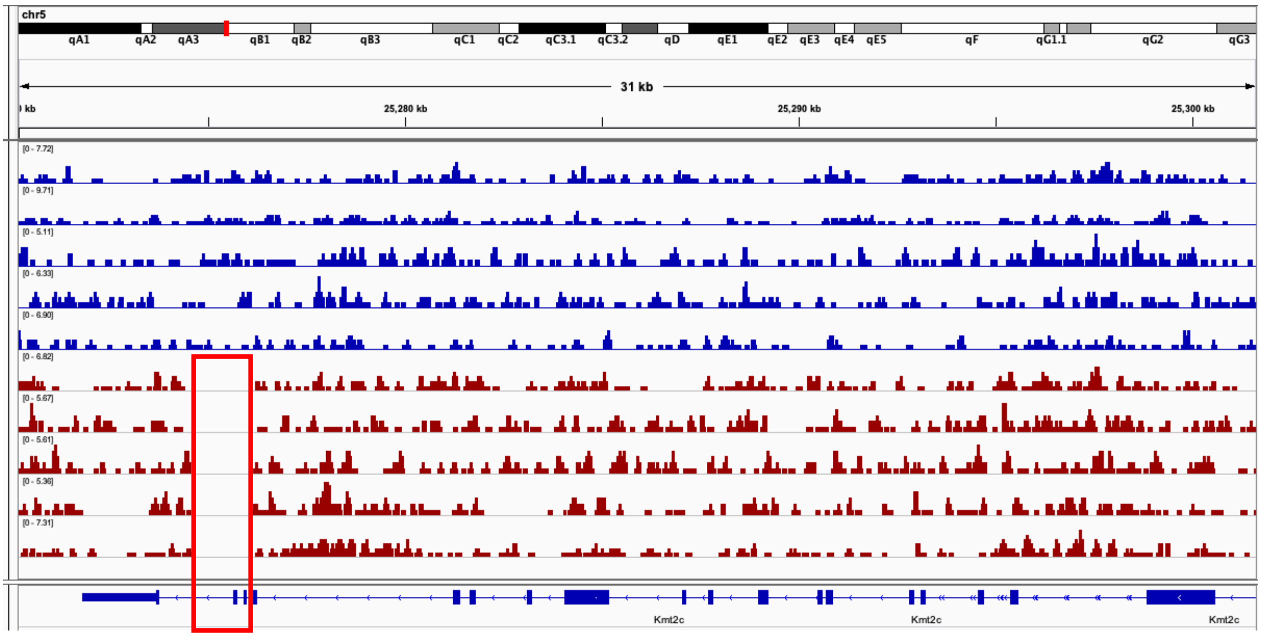

B

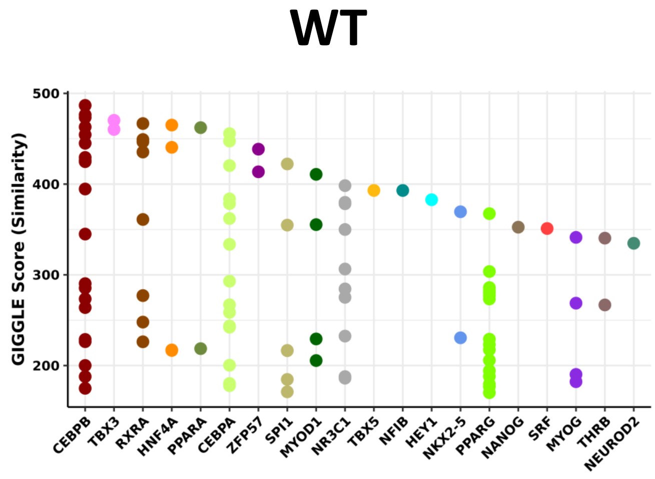

C

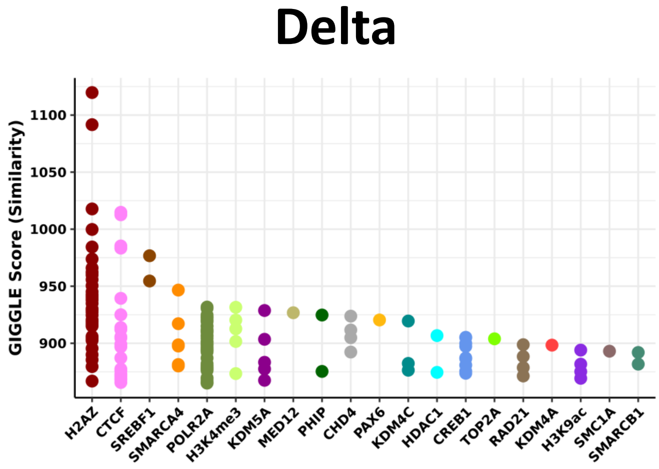

A. Gene tracks for each individual mouse across the MLL3 genomic locus. The deleted region of the MLL3 Delta allele, across exons 57-58, is highlighted in the red box. Tracks from wild-type mice are in blue, tracks from Delta mice are in red. B. H3K4me1 peaks which were found to be unique to the WT condition were submitted for Giggle score analysis to assess similarity with other published ChIP-seq factor binding datasets. C. Peaks which were found to be unique to the Delta condition were submitted for Giggle score analysis to assess similarity with other published ChIP-seq factor binding datasets. D. PCA analysis of all H3K4me3 ChIP-seq samples using Deeptools.

Figure 3 supplement

D

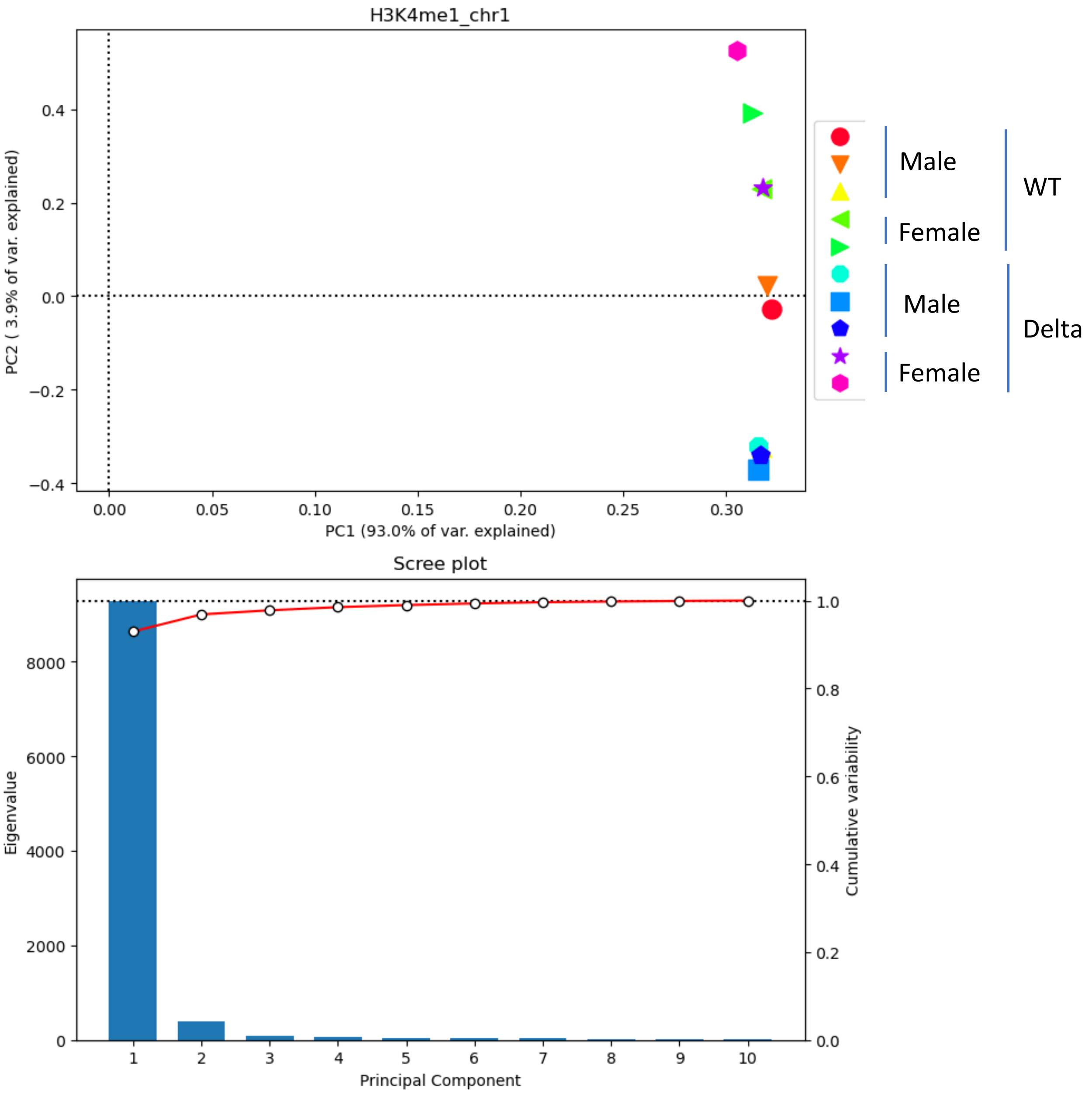

Figure 4. supplement

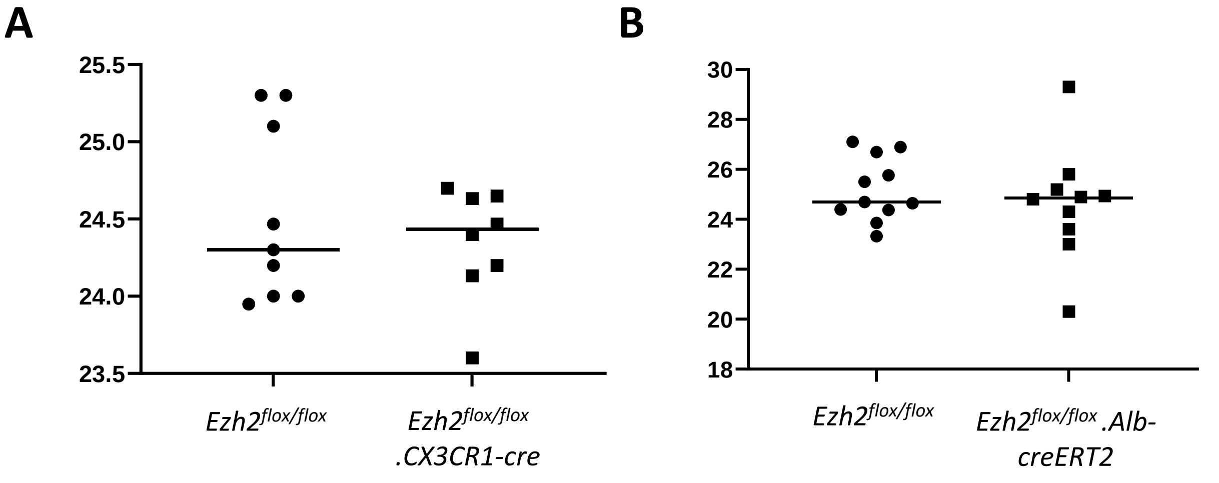

A. PECs were isolated from Ezh2 floxed Cx3cr1-Cre mice and Ezh2 floxed littermate controls, and placed into a lumicycle. Circadian oscillations in Per2::luc were measured for 3 days and period was calculated. Each data point represents the average of 3 technical replicates from an individual animal. B. Liver slices were taken from Ezh2 floxed AlbCreERT2 mice and Ezh2 floxed littermate controls, treated with tamoxifen for 5 days. Liver slices were taken 10 days after the final tamoxifen treatment and placed in a lumicycle. Circadian oscillations in Per2::luc were measured for 3 days and period was calculated. Each data point represents the average of 3 technical replicates from an individual animal.

Figure 5. supplement

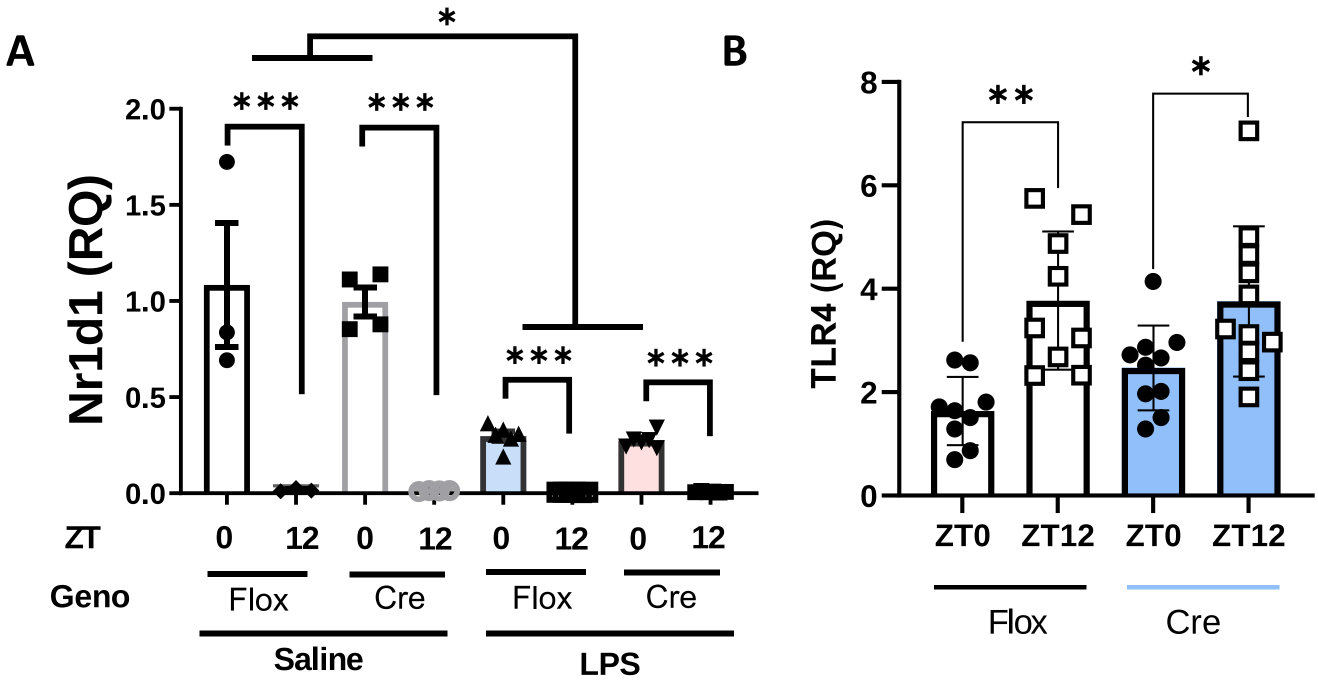

A. qPCR analysis of Reverba (NR1D1) expression in lung tissue from mice exposed to aerosolised LPS or saline control at ZT0 or ZT12. Lung tissue was harvested 5 hours after exposure. B. qPCR analysis of Tlr4 expression in lung tissue from mice exposed to aerosolised LPS or saline control at ZT0 or ZT12. Lung tissue was harvested 5 hours after exposure. As there was no effect of LPS exposure on Tlr4 expression, saline and LPS exposed data were pooled by timepoint and genotype.
